# Efficacy of Cathelicidin-Mimetic Antimicrobial Peptoids against Staphylococcus aureus

**DOI:** 10.1128/spectrum.00534-22

**Published:** 2022-04-25

**Authors:** Aaron B. Benjamin, Madeleine G. Moule, Maruti K. Didwania, Jonathan Hardy, Panatda Saenkham-Huntsinger, Preeti Sule, Josefine Eilsø Nielsen, Jennifer S. Lin, Christopher H. Contag, Annelise E. Barron, Jeffrey D. Cirillo

**Affiliations:** a Department of Microbial Pathogenesis and Immunology, Texas A&M Health Science Center, College Station, Texas, USA; b Department of Bioengineering, Stanford University grid.168010.egrid.471392.agrid.168010.e School of Medicine and of Engineering, Stanford, California, USA; c Institute for Quantitative Health Science and Engineering, Departments of Biomedical Engineering and Microbiology & Molecular Genetics, Michigan State University, East Lansing, Michigan, USA; d Institute of Immunology & Infection Research, School of Biological Sciences, University of Edinburgh, Edinburgh, United Kingdom; e Department of Science and Environment, Roskilde University, Roskilde, Denmark; University of Calgary

**Keywords:** *Staphylococcus aureus*, antimicrobial peptides, chemical synthesis

## Abstract

Staphylococcus aureus is one of the most common pathogens associated with infection in wounds. The current standard of care uses a combination of disinfection and drainage followed by conventional antibiotics such as methicillin. Methicillin and vancomycin resistance has rendered these treatments ineffective, often causing the reemergence of infection. This study examines the use of antimicrobial peptoids (sequence-specific poly-*N*-substituted glycines) designed to mimic naturally occurring cationic, amphipathic host defense peptides, as an alternative to conventional antibiotics. These peptoids also show efficient and fast (<30 min) killing of methicillin-susceptible S. aureus (MSSA) and methicillin-resistant S. aureus (MRSA) at low micromolar concentrations without having apparent cytotoxic side effects *in vivo*. Additionally, these novel peptoids show excellent efficacy against biofilm formation and detachment for both MSSA and MRSA. In comparison, conventional antibiotics were unable to detach or prevent formation of biofilms. One cationic 12mer, Peptoid 1, shows great promise, as it could prevent formation of and detach biofilms at concentrations as low as 1.6 μM. The use of a bioluminescent S. aureus murine incision wound model demonstrated clearance of infection in peptoid-treated mice within 8 days, conveying another advantage these peptoids have over conventional antibiotics. These results provide clear evidence of the potential for antimicrobial peptoids for the treatment of S. aureus wound infections.

**IMPORTANCE** Staphylococcus aureus resistance is a consistent problem with a large impact on the health care system. Infections with resistant S. aureus can cause serious adverse effects and can result in death. These antimicrobial peptoids show efficient killing of bacteria both as a biofilm and as free bacteria, often doing so in less than 30 min. As such, these antimicrobials have the potential to alleviate the burden that Staphylococcus infections have on the health care system and cause better outcomes for infected patients.

## INTRODUCTION

Antimicrobials are one of the most beneficial discoveries in all of medical history for humans. The use of these molecules has allowed for enhanced longevity of life through means such as livestock welfare and postsurgical infection prevention. Up until the 1970s, novel antibiotics were relatively abundant, with a consistent last-resort drug available for the worst antibiotic-resistant cases (1). Unfortunately, teixobactin is the only novel antibiotic discovered since the 1970s that has shown good activity against Gram-positive bacteria, including some drug-resistant microbes (2). While some drugs such as vancomycin were in use for 30 years before bacteria developed resistance to them (3, 4), it is inevitable that resistance develops over time to typically used classes of antibiotics.

Staphylococcus aureus is an example of bacteria found in the upper respiratory tract and skin flora for humans that have developed antibiotic resistance (5, 6). S. aureus is Gram-positive and a facultative anaerobe that can form biofilms. Initial treatments of S. aureus infections included penicillin and methicillin, but clinical practice has evolved to utilize other antibiotics such as vancomycin. Resistance in S. aureus has grown at a rapid pace since they were first treated with penicillin and methicillin. Resistance to these two antibiotics was reported at an incidence of around 0.4% of the total infections tested in 1960 (7). A study of resistance between 2009 and 2014 showed nearly all strains are resistant to penicillin G and 20% are resistant to methicillin (8). Additionally, this report showed that cultures display resistance to other antibiotics such as rifampin, tetracycline, and erythromycin. Vancomycin has been considered the go-to drug for those allergic to penicillin and for resistant cases; however, clinical isolates resistant to vancomycin began to appear as early as 1997 (4). As such, it is necessary to develop and pursue other classes of antimicrobials that are effective against all types of bacteria and to which bacteria do not develop resistance.

Nature has given us a variety of antimicrobial molecules that are part of the mammalian immune system and that act by diverse mechanisms. Many of these natural products are produced as a defense mechanism to protect against pathogens at barrier tissues. Some of these antimicrobials are peptides, commonly referred to as antimicrobial peptides (AMPs). As a general rule, these peptides tend to be less than 50 amino acids in length, are positively charged (net charge of +2 to +9), amphipathic and have about 50% hydrophobic residues (9, 10). α-helical AMPs have been studied extensively and this important group includes well-known AMPs such as the magainins and cathelicidins (9). These AMPs disrupt and pass through the cell membrane, either by pore formation or changes to lipid packing and dynamics, as seen by some magainins, protegrins and LL-37, which is the only human cathelicidin peptide (11–13). LL-37 is involved in numerous cellular functions, including defense against a broad range of microbial infections, and immunomodulation (14). LL-37 has been determined to not only target the membrane of microbes, but also has key intracellular effects, including ribosome flocculation, DNA and RNA binding (15), and a resulting rigidification of the cytoplasm (16). While AMPs have shown relative effectiveness against the development of bacterial resistance, they are easily degradable by proteases. As such, antimicrobial poly-*N*-substituted glycines (peptoids) present a solution to this problem. Peptoids are resistant to protease degradation due to the positioning of their side chain (R) groups on the nitrogen atom of the polypeptide backbone, rather than the α-carbon (17). In addition, they are easier and less expensive to synthesize than regular α-peptides. Similar to LL-37, Peptoid 1 has been shown to self-assemble in solution forming helical bundles (18). The mechanism of antibacterial and antifungal action for various AMP-mimetic peptoids has been investigated (15, 19, 20), and involves a combination of membrane disruption and profound intracellular effects, including DNA- and RNA-binding and ribosome flocculation, similar to what has been observed for LL-37 (15).

Peptoid 1 has previously been shown to have potent activity against a broad spectrum of bacteria, including resistant strains (18, 21–24), fungi (18), and viruses (25) while showing minimal cytotoxicity profiles against a number of mammalian cell lines (21, 25), and almost no toxicity to primary human cells grown at the air-liquid interface (25). Interestingly, Peptoid 1 has been shown to be extremely effective in preventing biofilm formation of Pseudomonas aeruginosa compared to standard antibiotics (23, 24), activity that is particularly relevant because of the tendency for wound infections to involve the growth of a strong biofilm. Peptoids also recently have been demonstrated to reduce bacterial counts in a murine model of an invasive S. aureus infection (26, 27). Remarkably, Peptoid 1 and several analogues have been proven to retain their anti-biofilm activity even in the context of polymicrobial infections comprised by clinical isolates of P. aeruginosa and S. aureus in host-mimicking conditions *in vitro* (18). In a murine abscess model, where Peptoid 1 was injected into the abscess, Peptoid 1 reduced both P. aeruginosa and S. aureus abscess size and bacterial load (18).

In this study, we investigated whether Peptoid 1 and two variants can be used for the topical treatment of wound infections, and against methicillin-resistant S. aureus strains. Initially, peptoids were evaluated against planktonic S. aureus, but were subsequently evaluated for their ability to prevent biofilm formation and facilitate biofilm detachment. The peptoids that were tested, especially Peptoid 1, show promise at killing both resistant and nonresistant S. aureus. Additionally, Peptoid 1 was able to prevent biofilm formation and to detach existing biofilms. Topical application of Peptoid 1 or Peptoid 1-C13_4mer_ in a PBS solution to a murine wound model showed a statistically significant reduction in bacterial counts compared to a saline-treated control group. These results demonstrate the potential of antimicrobial peptoids in the treatment of S. aureus biofilms and wound infections, as well as the potential for their use against other pathogenic bacteria. Taken together, results indicate that peptoids hold forth promise to be clinically translated as an important new class of broad-spectrum antibiotic drugs.

## RESULTS

### Peptoid design.

**FIG 1:**
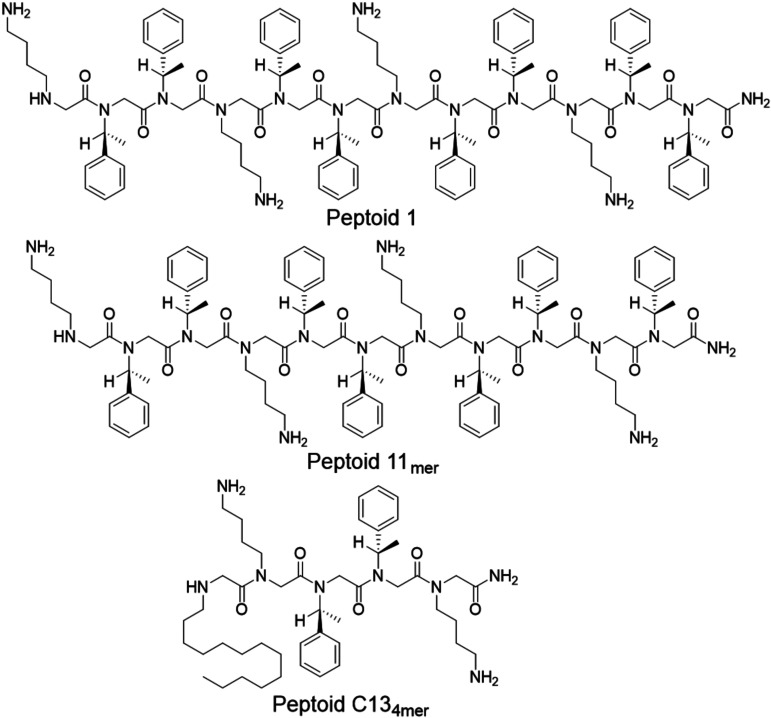
Chemical structures of the three different peptoids tested in this study. Peptoids are made up of three different monomers, *N*Lys, *N*spe, and *N*tridec.

Prior studies with the 12mer Peptoid 1 have shown great promise against planktonic S. aureus and biofilm-forming bacteria (18, 21, 23, 24, 27). The design of this peptoid was originally inspired by that of magainin-2, an antimicrobial peptide found in Xenopus laevis, and consists of four lysine-like monomers (*N*Lys) and eight phenylalanine-like monomers (*N*-1-S-phenylethyl, *N*spe) patterned to give the peptoid helical, water-soluble, and amphipathic properties (24). Biophysical mechanism of action studies by TEM and soft X-ray tomography showed that Peptoid 1 is a particularly good mimic of the human cathelicidin AMP LL-37 (17). Since Peptoid 1 has strong activity, two related peptoids, Peptoid 1-11_mer_ and Peptoid 1-C13_4mer_ were synthesized as mimics with slightly different properties (Table 1). These also mimic cationic AMPs like magainin-2; however, they have slightly different charges, amphipathicity, and length, allowing for better activity and selectivity against bacteria. Peptoid 1-11_mer_ has one fewer Nspe residue than Peptoid 1 and lower relative cytotoxicity compared to other peptoids (23, 28). Peptoid 1-C13_4mer_ is five residues long total, with its amino-terminal peptoid side chain being a 13-carbon-long *N*-alkyl tail, which has shown good activity against biofilms (23, 24). The long alkyl tail of Peptoid 1-C13_4mer_ promotes its self-assembly into ellipsoidal micelles, rather than helical bundles as for Peptoid 1 (18). The chemical structures of the peptoids used in this study are depicted in Fig. 1, with their respective monomer sequences given in Table 1.

**TABLE 1:** Antimicrobial activity of peptoids and LL-37 against MRSA, Xen 29, and Xen 36

ID	Sequence	TFA salt			Chloride salt		
		MRSA (μM)	Xen 29 (μM)	Xen 36 (μM)	MRSA (μM)	Xen 29 (μM)	Xen 36 (μM)
Peptoid 1	H-(*N*Lys-*N*spe-*N*spe)_4_-NH_2_	< 0.78	< 0.78	12.5	3.125	ND^*a*^	1.56
Peptoid 1-C13_4mer_	H-*N*tridec-*N*Lys-*N*spe-*N*spe-*N*Lys-NH_2_	12.5	12.5	25	3.125	ND	3.125
Peptoid 1-11_mer_	H-(*N*Lys-*N*spe-*N*spe)_3_-*N*Lys-*N*spe-NH_2_	50	25	25	25	ND	12.5
LL-37	LLGDFFRKSKEKIGKEFKRIVQRIKDFLRNLVPRTES	> 100	> 100	50	ND	ND	ND

a ND, not done.

### Determination of peptoid effectiveness against S. aureus infections.

MICs for these peptoids were evaluated against strains of S. aureus (Table 1). The TFA salt of Peptoid 1 had an MIC of <0.78 μM for both MRSA and Xen29, and 12.5 μM for Xen36. Peptoid 1-C13_4mer_ had MICs of 12.5 μM for MRSA and Xen29, and 25 μM for Xen36, while Peptoid 1-11_mer_ had MICs of 50 μM for MRSA, and 25 μM for Xen29 and Xen36. When neutralized into the chloride salt, MICs against MRSA were 3.125 μM for Peptoid 1 and Peptoid 1-C13_4mer_, and 25 μM for Peptoid 1-11_mer_, while Xen36 had MICs of 1.56 μM, 3.125 μM, and 12.5 μM for Peptoid 1, Peptoid 1-C13_4mer_, and Peptoid 1-11_mer_, respectively. To compare, antibiotics such as amoxicillin and ciprofloxacin had little effect on MRSA, with an MIC >100 μM (Table 2). Both antibiotics were highly effective against the methicillin-susceptible strain, Xen29, yielding MICs <0.78 μM. Ciprofloxacin also showed good effectiveness against Xen36 with an MIC of 1.6 μM, however, amoxicillin was ineffective with an MIC >100 μM. While these antibiotics are typically used for nonresistant S. aureus, Vancomycin and Daptomycin are often used for drug-resistant strains. Daptomycin was minimally effective, with MICs of 50 μM and 25 μM for MRSA and Xen36, respectively. Vancomycin, the “gold-standard” for drug-resistant S. aureus, had MICs of 0.195 μM for MRSA and 0.39 μM for Xen36.

**TABLE 2:** Antimicrobial activity of conventional antibiotics against MRSA, Xen 29, and Xen 36

Antibiotic	Molecular formula	MRSA (μM)	Xen 29 (μM)	Xen 36 (μM)
Amoxicillin	C_16_H_19_N_3_O_5_S	> 100	< 0.78	> 100
Ciprofloxacin	C_17_H_18_FN_3_O_3_	> 100	< 0.78	1.6
Vancomycin	C_66_H_75_Cl_2_N_9_O_24_	0.195	ND^*a*^	0.39
Daptomycin	C_72_H_101_N_17_O_26_	50	ND	25

a ND, not done.

**FIG 2:**
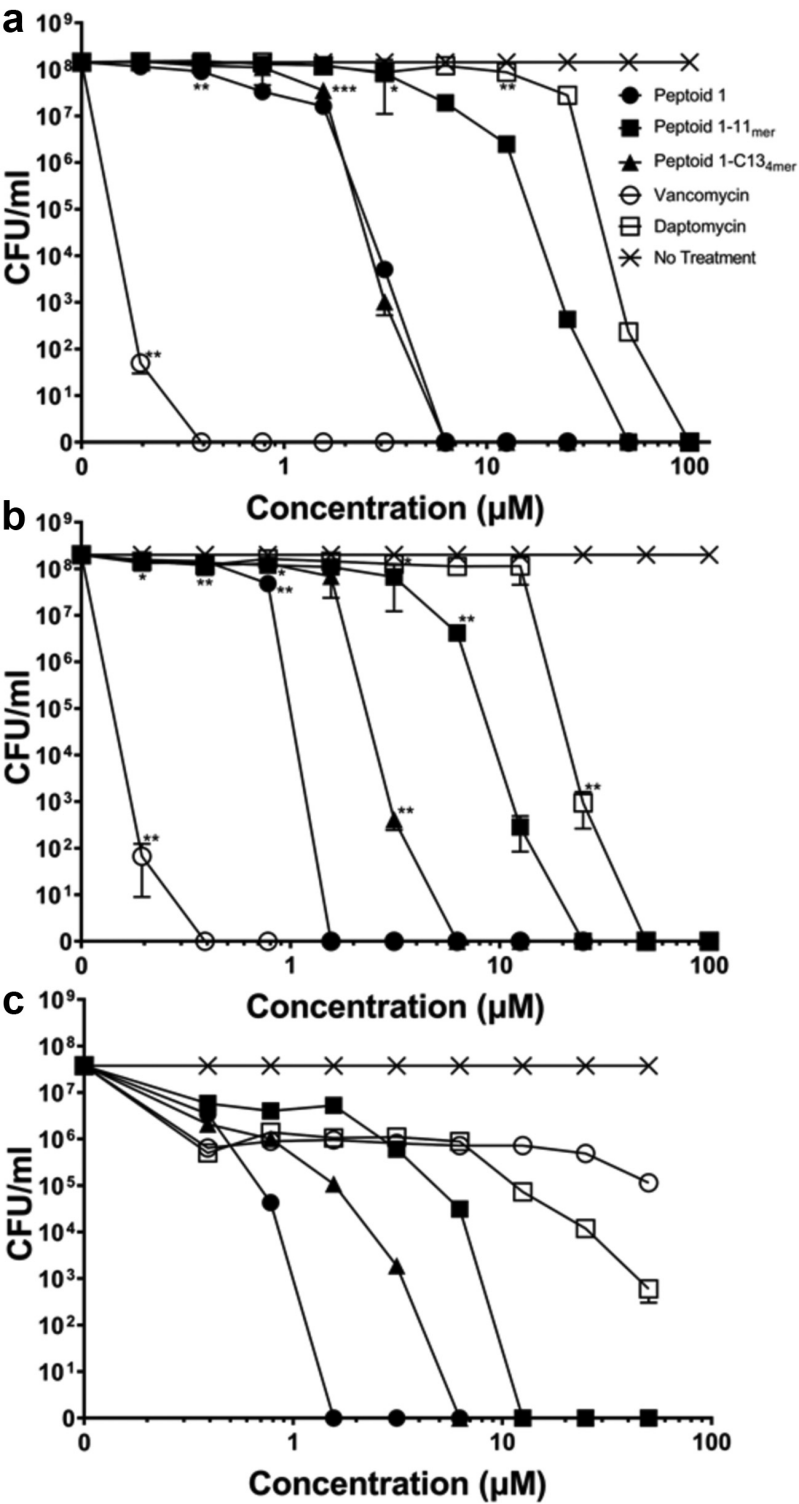
CFU/ for drug-resistant/drug-sensitive strains of S. aureus (a) Xen36, (b) MRSA 252, and (c) Xen 29 with Peptoid 1, Peptoid 1-C13_4mer_, Peptoid 1-11_mer_, and traditionally used antibiotics vancomycin and daptomycin at various concentrations on LB plates after 24 h. (a) For Xen36, vancomycin was significantly different from the no treatment control at 0.19 μM and above, while Peptoid 1 was significant starting at 0.78 μM and above. Peptoid 1-C13_4mer_ showed significance starting at a concentration of 3.125 μM. Peptoid 1-11_mer_ showed significance at 0.19 μM but didn’t show significance again until 0.78 μM. From here, Peptoid 1-11_mer_ didn’t show significance again until 6.25 μM. Similarly, daptomycin showed significance at 0.39 μM but didn’t show significance again until 3.125 μM. Daptomycin remained statistically significant until 6.5 μM, but only regained significance compared to the no treatment control from 25 μM onward. (b) Vancomycin once again showed significance at 0.39 μM compared to the no treatment control, this time for the clinical MRSA 252 strain. Peptoid 1 showed significance starting at 0.78 μM, while Peptoid 1-C13_4mer_ showed significance starting at 1.56 μM. Peptoid 1-11_mer_ showed significance 6.25 μM and daptomycin showed significance starting at 12.5 μM. (c) All treatments showed significance starting at 0.39 μM for Xen29. Data points are represented as means using four replicates. Error is shown in ± standard deviation (SD). Statistics were performed using 2-way ANOVA, comparing each antimicrobial to the no treatment control. *P* values are: <0.0001 = ****, between 0.0001 and 0.001 = ***, between 0.001 and 0.01 = **, and between 0.01 and 0.05 = *.

Since MRSA is a drug-resistant strain, it was necessary to determine the concentration necessary for complete killing for these peptoids. After colonies had been counted, Peptoid 1 provided complete killing at a concentration greater than 1.56 μM and 6.25 μM for Xen36 and MRSA, respectively (Fig. 2a and b). Peptoid 1 was effective against the drug-susceptible, Xen29 strain, showing zero living CFU/mL at Peptoid 1 concentrations higher than 1.56 μM. For Peptoid 1-C13_4mer_, all three strains showed complete killing at a concentration of 6.25 μM and above (Fig. 2). Peptoid 1-11_mer_ was slightly more effective at complete killing in Xen29, eliminating all bacteria at concentrations of 12.5 μM and above, while doing so for the drug-resistant strains only at 25 μM and above (Fig. 2). CFU were calculated for vancomycin and daptomycin since they are utilized against drug-resistant S. aureus. Daptomycin only showed complete killing above 50 μM for Xen36, and 100 μM for MRSA 252 (Fig. 2a, b). In contrast to the poor killing by daptomycin, vancomycin showed complete killing for both strains above a concentration of 0.39 μM (Fig. 2a, b). Interestingly, Xen29 did not show complete killing for either antibiotic up to 50 μM, whereas it showed nearly complete killing for daptomycin at this concentration (Fig. 2c). All treatments showed a statistically significant decrease (*P* < 0.01) at all concentrations compared to nontreated cells for the Xen29 strain. Statistically, vancomycin showed a significant decrease (*P* < 0.01) in CFU compared to nontreated cells from 0.39 μM and upward for the Xen36 strain and MRSA 252. In comparison, Peptoid 1 showed statistically significant decrease (*P* < 0.01) in CFU compared to nontreated cells from 0.78 μM and higher in MRSA 252 and 1.56 μM in Xen36.

### Cytotoxicity of peptoids.

**FIG 3:**
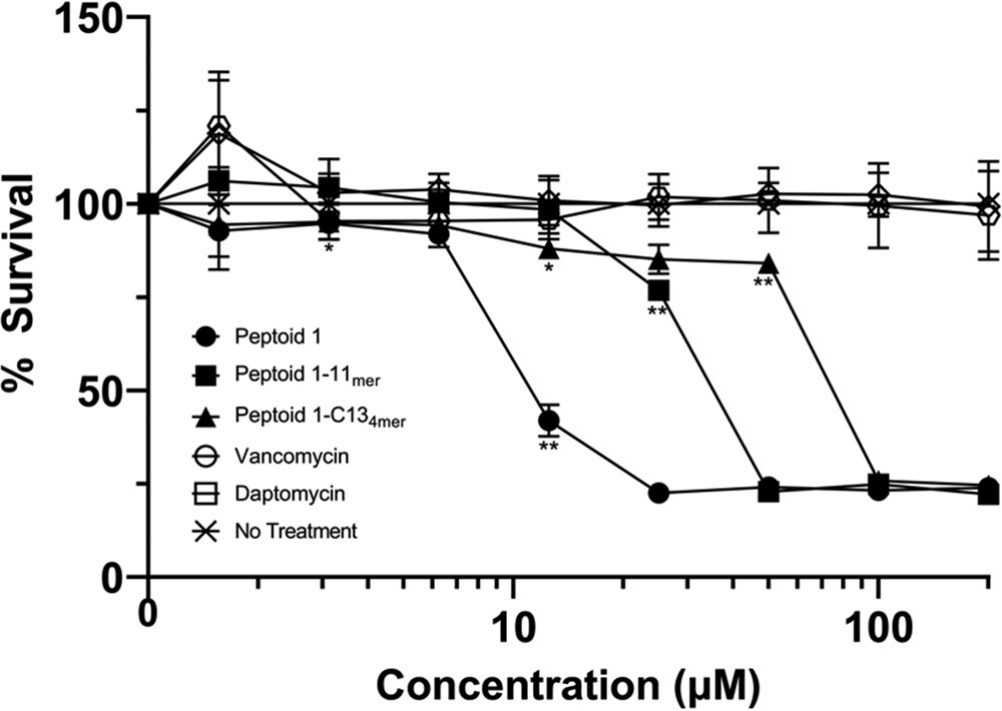
Cytotoxicity of peptoids and traditionally used antibiotics in J774A.1 cells in DMEM media. The half maximal inhibitory concentration (IC_50_) was calculated as the concentration at which 50% of the cells were killed. Data points are represented as means using three replicates. Error bars are represented as ± standard deviation (SD). Peptoid 1 showed significance compared to the no treatment control at 3.125 μM and from 12.5 μM and upward. Peptoid 1-C13_4mer_ was significant at 12.5 μM initially and from 50 μM and upward, while Peptoid 1-11_mer_ showed significance at 25 μM and above. Statistics were performed using 2-way ANOVA, comparing each antimicrobial to the no treatment control. *P* values are: <0.0001 = ****, between 0.0001 and 0.001 = ***, between 0.001 and 0.01 = **, and between 0.01 and 0.05 = *.

Cytotoxicity of these peptoids to macrophages by comparison to conventional antibiotics was determined using J774A.1 mouse macrophages and 3T3 mouse fibroblasts. Incubation with peptoids allowed for the calculation of J774A.1 cells showed a half-maximal inhibitory concentration (IC_50_) of 12.5 μM for Peptoid 1, while Peptoid 1-11_mer_ had an IC_50_ of 50 μM (Fig. 3). Peptoid 1-C13_4mer_ had the highest IC_50_, at 100 μM, while both vancomycin and daptomycin had an IC_50_ greater than the highest concentration tested at 256 μM. The 3T3 cells showed an IC_50_ for Peptoid 1 of 15 μM, while Peptoid 1-11_mer_ had an IC_50_ of about 45 μM (Fig. S1). Peptoid 1-C13_4mer_ had the highest IC_50_ for the 3T3 cells at over 100 μM. In general, when AMPs and their mimics are tested against immortalized cell lines such as these mouse macrophages, they show apparently higher toxicities than are seen against primary human cells (29).

### Effect of peptoids on biofilm formation and detachment.

**FIG 4:**
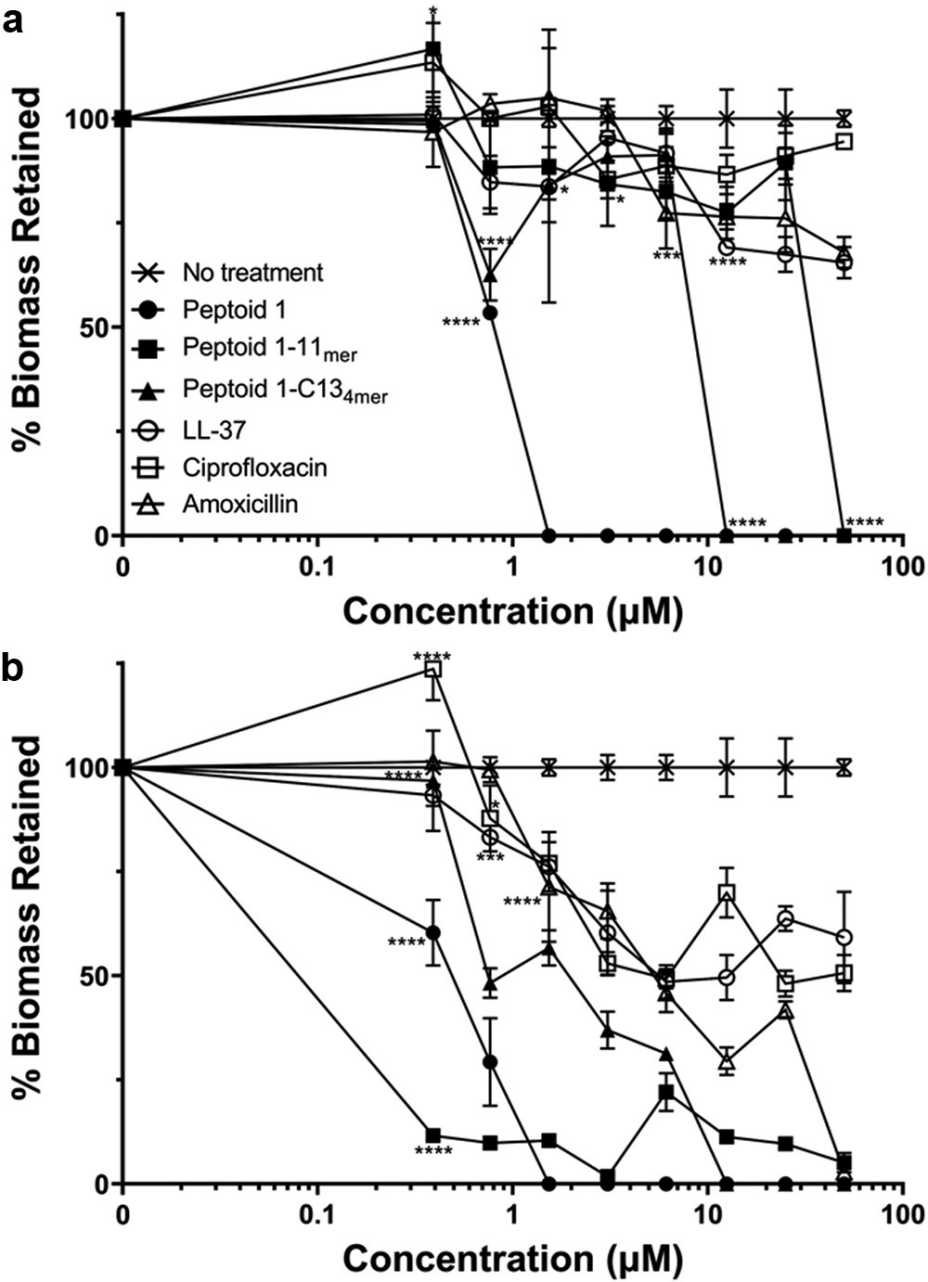
(a) Measurement of MRSA biofilm formation after 24 h in the presence of antimicrobial peptoids (AMPs) and conventional antibiotics. Prevention of biofilm formation is plotted as percent biomass analyzed by crystal violet assay. Data points are represented as means using three replicates. Error bars are represented as ± standard deviation (SD). Peptoid 1, Peptoid 1-11_mer_, and Peptoid 1-C13_4mer_ showed a significant change in biofilm formation compared to the no treatment control from 0.39 μM and upward. LL-37 showed significance starting at 0.78 μM while amoxicillin showed significance starting at 1.56 μM. Ciprofloxacin showed a significant increase in biofilm formation at 0.39 μM, however, showed a significant decrease at concentrations of 0.78 μM and up. (b) Measurement of MRSA biofilm detachment after 24 h by AMPs and conventional antibiotics. Detachment is plotted as percent biomass of established biofilms after treatment with antimicrobials. Data points are represented as means using three replicates. Error bars are represented as ± SD. Peptoid 1 showed significance compared to the no treatment control at concentrations of 0.78 μM and above, while Peptoid 1-C13_4mer_ showed initial significance at 0.78 μM to 1.56 μM, however, did not show significance until 12.5 μM and above. Peptoid 1-11_mer_ showed a statistical increase in biofilm formation at 0.39 μM but showed a significant decrease from 3.125 μM to 12.5 μM and at 50 μM. LL-37 showed significance at 1.56 μM but not again until 12.5 μM and above. Amoxicillin showed significant difference from the no treatment control starting at a concentration of 6.35 μM, however, ciprofloxacin did not show a significant difference at any concentration. Values for both experiments were calculated as OD_490_ (Treated)/OD_490_ (Untreated) * 100. Statistics were performed using 2-way ANOVA, comparing each antimicrobial to the no treatment control. *P* values are: <0.0001 = ****, between 0.0001 and 0.001 = ***, between 0.001 and 0.01 = **, and between 0.01 and 0.05 = *.

Biofilm formation is often a problem for drug effectiveness and ability of a patient to eliminate a pathogen like S. aureus. To test whether these peptoids could prevent biofilm formation, MRSA was cultured with various concentrations of peptoid, antimicrobial peptide, or antibiotic. Results from this assay showed a complete reduction of biofilm formation at 1.6 μM Peptoid 1 and at 12.5 μM for Peptoid 1-C13_4mer_ (Fig. 4a). Peptoid 1-11_mer_ was unable to prevent biofilm formation up to 50 μM, however, it reduced biofilm mass by 95%. In comparison, amoxicillin was able to reduce biofilm formation by 97% percent at 50 μM and ciprofloxacin only reduced biofilm formation by 49%. The human cathelicidn LL-37 was only able to reduce biofilm formation by 41%. The methicillin-susceptible strain, Xen29, showed complete reduction of biofilm formation by Peptoid 1 at 1.6 μM as well (Fig. S2a); however, Xen36 only showed a 74% reduction (Fig. S2b). Peptoid 1-C13_4mer_ showed complete prevention of biofilm formation at 12.5 μM in Xen29 and a reduction of 40% by 50 μM in Xen36. In Xen36, Peptoid 1-11_mer_ reduced biofilm formation by 70% at 50 μM, however, Peptoid 1-11_mer_ did not appear to influence biofilm formation in Xen29. This was interesting, as this pattern appeared to be similar for LL-37. In Xen36, there was a 60% reduction and no apparent reduction in Xen29. Ciprofloxacin prevented biofilm formation by 0.9 μM in Xen29 and reduced formation of biofilms by 75% above a concentration of 1.5 μM in Xen36. Similarly, amoxicillin prevented formation of biofilms in Xen29 by 0.39 μM, however, there was minimal reduction of biofilm by a concentration of 50 μM in Xen36.

While prevention of biofilm formation is important at the beginning of infection, it is also necessary to reduce biofilm that has already formed, especially in cases which have been left untreated. As such, a detachment assay was used by measuring biomass of biofilm remaining after treatment with peptoids, AMPs, or antibiotics. Peptoid 1 was able to detach all biofilm by a concentration of 1.6 μM in MRSA (Fig. 4b) and Xen29 (Fig. S3a), however, it was only able to detach up about 75–80% of biofilms at 50 μM in Xen36 (Fig. S3b7). Interestingly, complete biofilm detachment in MRSA was seen by Peptoid 1-C13_4mer_ at 12.5 μM and Peptoid 1-11_mer_ at 50 μM. In Xen29, on the other hand, detachment reached 35% for Peptoid 1-C13_4mer_ and 25% for Peptoid 1-11_mer_ by 50 μM. Xen36 showed more promising results for Peptoid 1-C13_4mer_ with reduction of biofilm by 70% at 50 μM; however, Peptoid 1-11_mer_ fared about the same, with a reduction of only 30% at that same concentration. LL-37 detached biofilms poorly, with 35%, 22%, and 20% for MRSA, Xen29, and Xen36, respectively. Amoxicillin eliminated all biofilm by 50 μM in Xen29, however, only resulted in 33% reduction in MRSA and 8% in Xen36 at that same concentration. Detachment of biofilms in ciprofloxacin was poor for MRSA, 6%, and Xen29, 15%; however it was able to reduce about 60% of Xen36 biofilms by 50 μM. These experiments showed the possibility of these peptoids to be useful *in vitro*, so it was necessary to validate these results *in vivo* as well. Since Peptoid 1 and Peptoid 1-C13_4mer_ appeared to be the most promising candidates based on the data presented here together with previous data showing activity against S. aureus abscesses when injected in a mouse model (18), these were selected to move on to further studies in mice using a topical application.

### Efficacy of peptoids in murine incision wound model.

**FIG 5:**
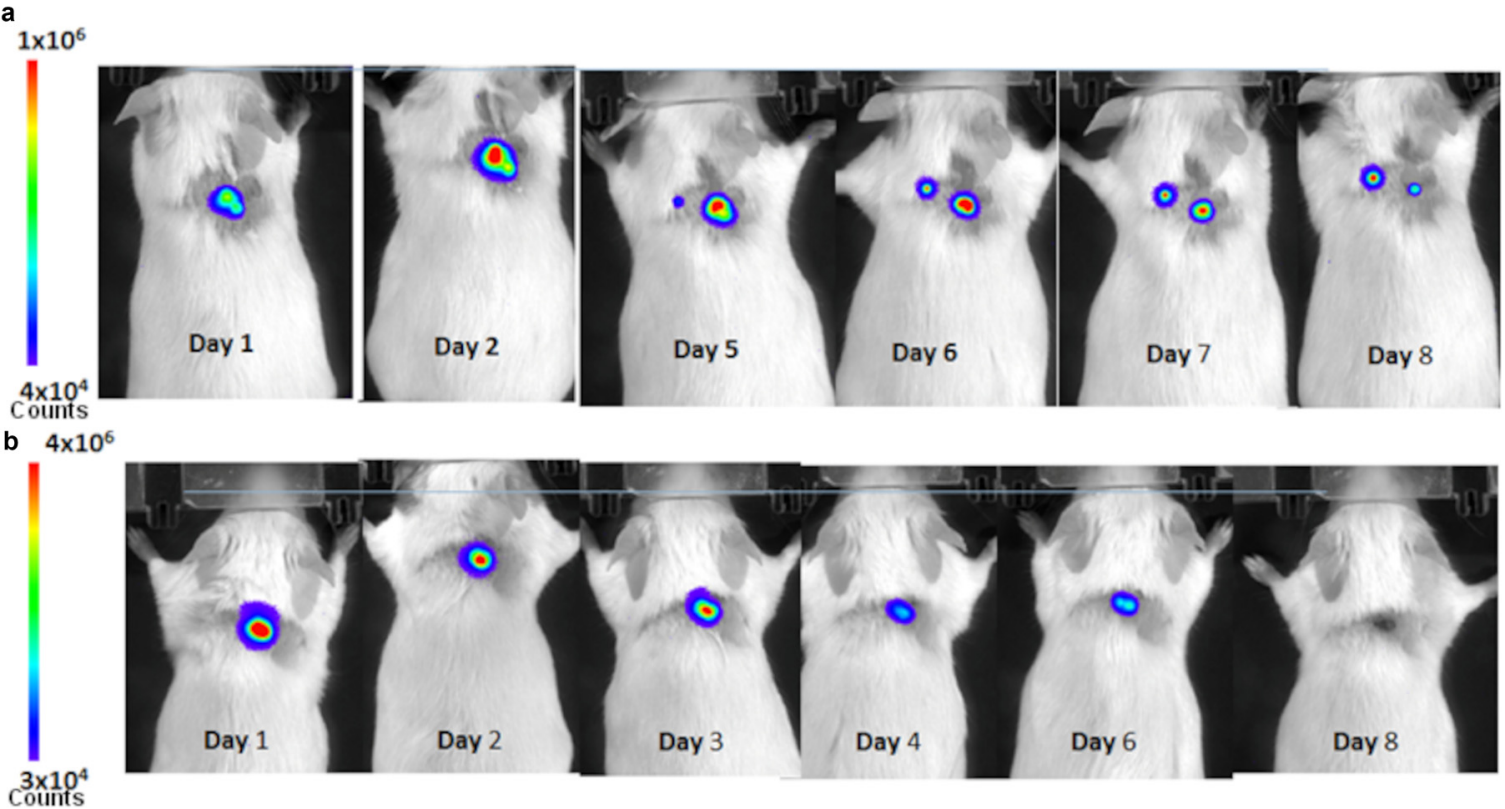
*In vivo* bioluminescence imaging. Bioluminescent Staphylococcus aureus, Xen36, was injected into the incision wounds. (a) Control mouse treated with phosphate-buffered saline (PBS) showed bioluminescence after 8 days, indicating the infection was still present. Note that the infection has migrated to a secondary site in the untreated animal by day five. (b) Mouse whose infected wound was treated with 100 μM Peptoid 1 in PBS showed negligible bioluminescence at day eight.

**FIG 6:**
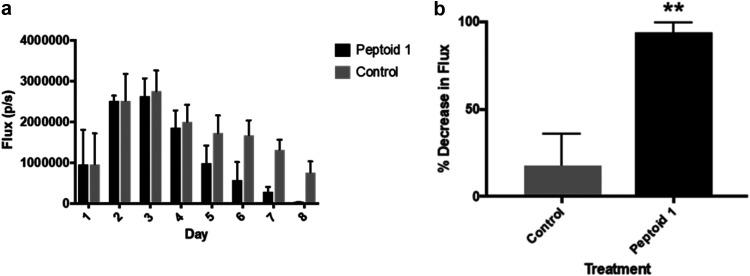
(a) *In vivo* bioluminescence imaging flux results versus day for the murine wound model experiment. A sample of four mice were taken for flux determination each day. (b) *In vivo* bioluminescence imaging % decrease in flux on day eight. Results were statistically significant with *P* = 0.05.

In order to see the effectiveness of these peptoids *in vivo*, mice were injected with bioluminescent S. aureus Xen36 via a murine incision wound model. Mice were treated with a high dosage of peptoid in PBS due to the inefficiency of drug delivery through the scab of the wound. After 1 day, both control and Peptoid 1-treated mice showed flux of about 10^6^ photons/second (Fig. 5). After day 3, flux in the Peptoid 1 treated mice were lower than the initial injection. By day 8, control mice showed two main areas of flux, while Peptoid 1-treated mice showed minimal flux. Upon further investigation, it became clear that the mice had flux from deeper within the wound (Fig. S4). In addition to the deeper flux seen in these mice, treatment with peptoid when dissolved in water required 8× as much peptoid than when treated with peptoid dissolved in PBS, likely due to reduced peptoid self-assembly and solubility in the absence of salt to screen peptoid-peptoid intermolecular interactions (Fig.  S5). When taking this all into account and quantifying, bacteria clearance in Peptoid 1 treated mice reduced flux by 90%, while the PBS control reduced the flux by 15% (Fig. 6). Despite the high concentration of peptoid used, mice did not appear to suffer any adverse side effects from treatment, likely because the peptoids are less cytotoxic to primary murine epithelial cells than they are to immortalized murine macrophages. Peptoid 1-C13_4mer_ showed similar results at 8 days postinfection, with minimal flux, while control mice still showed flux, although it had reduced somewhat (Fig. S6). Interestingly, both sets of control (untreated) mice using in the studies for Peptoid 1 and Peptoid 1-C13_4mer_ showed a secondary site of flux form by day 8.

### Timeframe of activity for peptoids versus traditional antibiotics.

**FIG 7:**
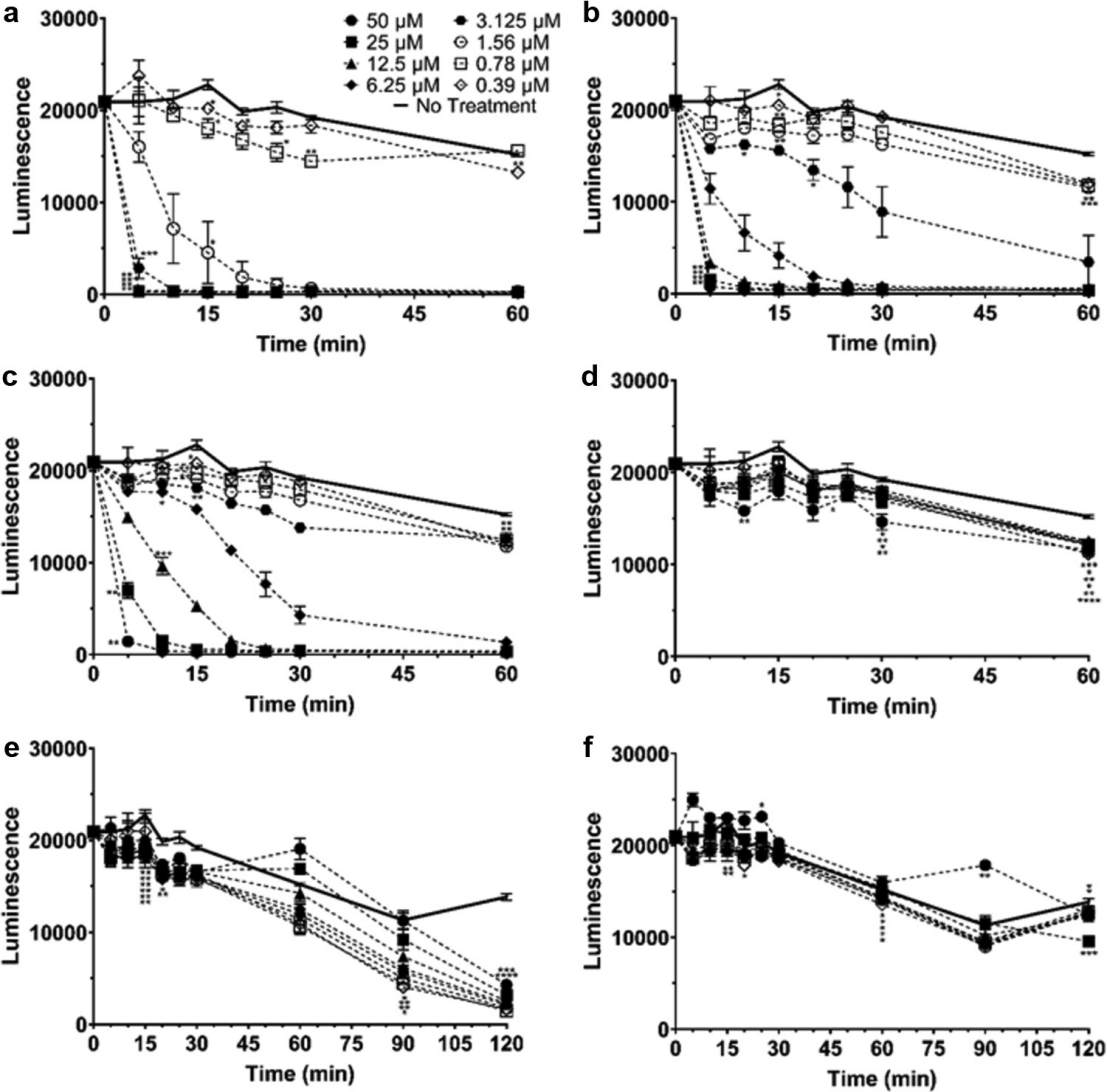
Kinetics of killing over 60 to 120 min in LB for Xen29 with (a) Peptoid 1, (b) Peptoid 1-C13_4mer_, (c) Peptoid 1-11_mer_, (d) LL-37, (e) vancomycin, and (f) daptomycin. Luminescence was measured over 60 min, at 5-min intervals up to 30 min. Concentrations ranging from 0.39 to 50 μM were tested for each peptoid, peptide, and antibiotic. (a) For Peptoid 1, concentrations of 3.125 μM and higher showed a significant decrease compared to the no treatment control for all time points. For 1.56 μM Peptoid 1, showed significance starting at 15 min. Peptoid 1 showed a significant decrease for 0.78 μM at 15 min and once again from 25 to 30 min while it showed a significant decrease for 0.39 μM from 15 to 20 min and once again at 60 min. (b) for Peptoid 1-C13_4mer_, concentrations of 6.125 μM and up were significantly different than the no treatment control for all time points. At 3.125 μM, Peptoid 1-C13_4mer_ showed a significant decrease from 10 to 25 min, while 1.56 μM showed significance at 15 min and from 25 min onward. Both 0.78 μM and 0.39 μM show significance at 15 min and once again at 60 min. (c) For Peptoid 1-11_mer_, significance was seen for 25 μM and 50 μM for all time points tested. At 6.25 μM and 12.5 μM, significance began after 10 min, while for 3.125 μM and 1.56 μM, significance was seen after 15 min. 0.78 μM and 0.39 μM showed a significant decrease at 15 min as well, however, they both didn’t show significance again until 60 min. (d) LL-37 showed a significant decrease for 50 μM and 25 μM starting at 10 min onward, however, 50 μM was not significant at 20 min while 25 μM was not significant at 30 min. 12.5 μM LL-37 showed significance at 15 min and from 30 min onward, while 6.25 μM only showed significance at 15 min and 60 min. 3.125 μM was significant only at 60 min, while 1.56 μM and 0.39 μM were significant from 30 min onwards. 0.78 μM was significant at 15, 25 and 60 min. (e) Vancomycin showed significance for 50 μM and 25 μM from 15 min to 30 min, and again at 120 min. At 12.5 μM, vancomycin showed significance from 15 to 30 min, and from 90 min onwards. At 6.25 μM and 0.78 μM, vancomycin showed significance from 15 min onward. 1.56 μM showed significance from 15 min to 30 min and from 90 min onward while both 3.125 μM and 0.39 μM were significant from 20 min to 30 min and from 90 min onward. (f) Daptomycin showed significant increases for 50 μM at 25 min and 90 min, however, showed a decreases compared to the no treatment control at 120 min. Both 25 μM and 12.5 μM daptomycin showed a significant decrease at 120 min, while 6.25 μM daptomycin showed an initial decrease at 15 min, and again at 60 min. 3.125 μM and 0.78 μM showed a significant decrease at 60 min, while 1.56 μM showed a significant decrease at 15 min and again at 60 min. 0.39 μM showed a significant decrease at 30 min and 60 min. All data points are represented as means using three replicates. Error bars are represented as ± standard deviation (SD). Statistics were performed using 2-way ANOVA, comparing each concentration over time to the no treatment control. *P* values are: <0.0001 = ****, between 0.0001 and 0.001 = ***, between 0.001 and 0.01 = **, and between 0.01 and 0.05 = *.

**FIG 8:**
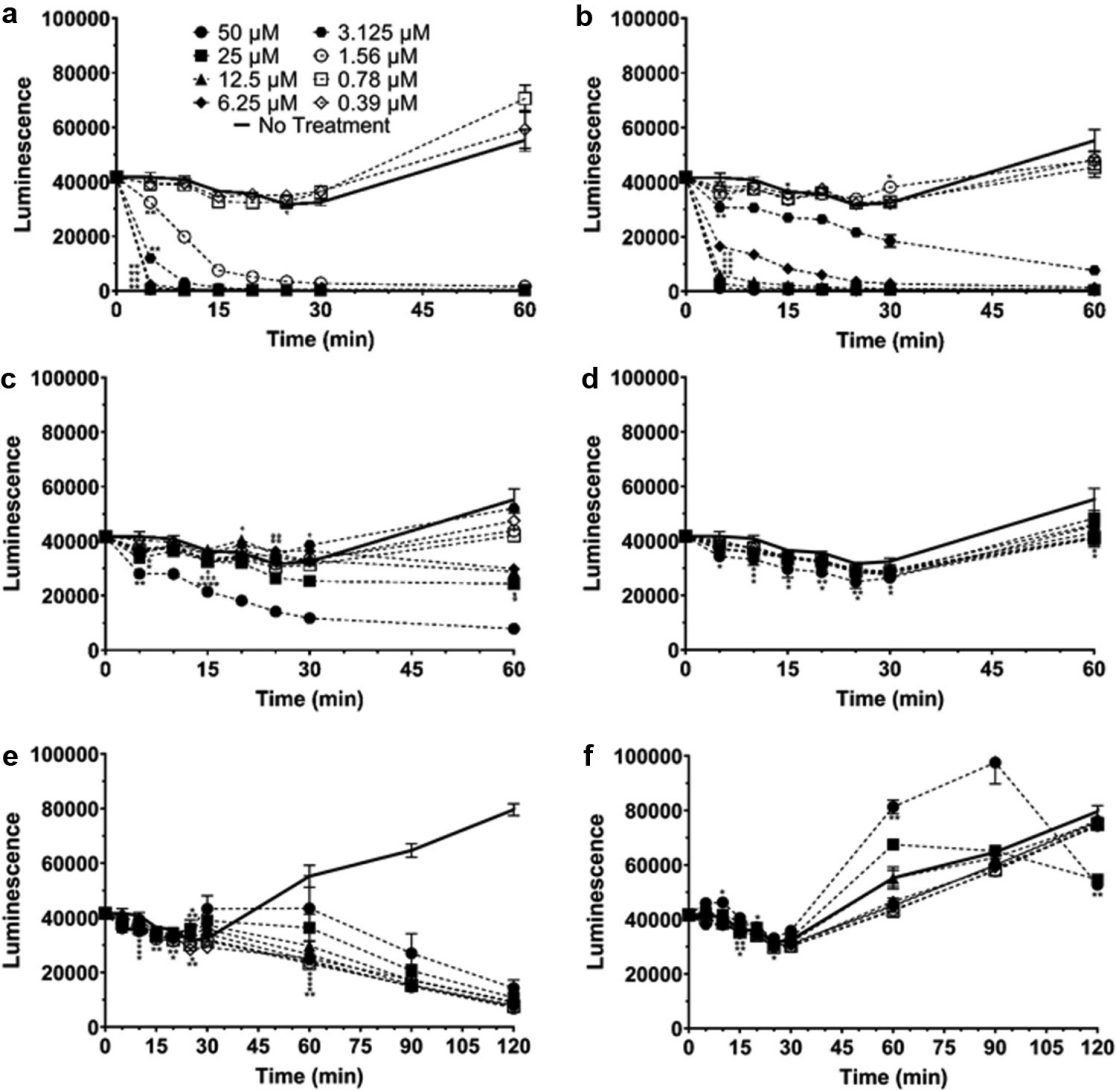
Kinetics of killing over 60 to 120 min for Xen36 with (a) Peptoid 1, (b) Peptoid 1-C13_4mer_, (c) Peptoid 1-11_mer_, (d) LL-37, (e) vancomycin, and (f) daptomycin. Luminescence was measured over 60 min, at 5-min intervals up to 30 min. Concentrations ranging from 0.39 to 50 μM were tested for each peptoid, peptide, and antibiotic. (a) Peptoid 1 showed a significant decrease compared to the no treatment control at all time points for 1.56 μM and above, while showing significance for 0.78 μM at 25 min. (b) Peptoid 1-C13_4mer_ showed significance for all time points at 3.125 μM and higher. 1.56 μM Peptoid 1-C13_4mer_ showed a significant decrease at 5 min, but showed a significant increase at 30 min, while 0.39 μM showed significance at 15 min. (c) Peptoid 1-11_mer_ showed significance for 25 μM and 50 μM for all time points tested, while 12.5 μM showed significance at 5, 20, and 60 min. 6.25 μM showed a significant decrease initially at 15 min, an increase at 25 min, and a decrease again at 60 min. 3.125 μM showed a statistical increase at 30 min, while 1.56 μM Peptoid 1-11_mer_ was significant at 15 min. 0.78 μM was significant at both 15 and 20 min. (d) LL-37 showed significance for 50 μM at 5, 10, and 30 min, while showing significance for 25 μM and 12.5 μM at 10, 20, and 25 min. Both 6.25 μM and 0.78 μM LL-37 showed a significant decrease compared to the no treatment control from 15 to 25 min, while 3.125 μM showed a significant decrease at 10, 25, and 60 min. 1.56 μM showed significance at 25 and 60 min, while 0.39 μM LL-37 showed significance from 30 min onward. (e) For vancomycin, 50 μM showed a significant decrease starting at 60 min, while 25 μM showed a significant increase from 25 to 30 min and a significant decrease from 60 min onward. 12.5 μM vancomycin showed a statistically significant decrease initially from 10 min to 15 min, however, it showed an increase at 25 min, and subsequent decrease at 60 min onward. 6.25 μM showed significance from 10 to 25 min and again after 60 min onward. Vancomycin showed significance at 3.125 μM from 10 to 20 min and again from 60 min onward, while showing significance for 1.56 μM at 15 and 25 min, and from 60 min onward. 0.78 μM showed significance at 10 and 20 min, and from 60 min onward, while 0.39 μM vancomycin showed significance from 15 to 25 min and from 60 min onward. (f) Daptomycin showed a significant increase initially for 50 μM at 10 min and again from 60 min to 90 min, with a significant decrease at 120 min. 25 μM showed significance at 15 min and again at 120 min, while 3.125 μM and 0.39 μM showed significance only at 15 min. 1.56 μM showed significance at 20 min and 0.78 μM showed significance at 25 min. All data points are represented as means using three replicates. Error bars are represented as ± standard deviation (SD). Statistics were performed using 2-way ANOVA, comparing each concentration over time to the no treatment control. *P* values are: <0.0001 = ****, between 0.0001 and 0.001 = ***, between 0.001 and 0.01 = **, and between 0.01 and 0.05 = *.

Since these peptoids showed promise as potential therapeutics, the time frame necessary to kill S. aureus was tested for these and traditionally used antibiotics (Fig. S7). Bioluminescence was measured for S. aureus incubated with peptoids at concentrations ranging from 0.39–50 μM for a total of 60 min, taking readings every 5 min for the first 30 min. Interestingly, it appeared that Peptoid 1 was able to kill Xen29 to background levels of luminescence in 30 min at concentrations of 1.5 μM and higher (Fig. 7a). Peptoid 1-C13_4mer_ was able to kill Xen29 to background levels at concentrations of 6.25 μM and greater by 30 min, while Peptoid 1-11_mer_ was able to achieve background levels at concentrations higher than 12.5 μM (Fig. 7b and c). LL-37, vancomycin, and daptomycin were unable to kill Xen29 to background levels by 30 min, however, vancomycin and daptomycin showed lower levels of luminescence than untreated bacteria at time points after 60 min (Fig. 7c-f). As such, all three peptoids took considerably less time than typically used antibiotics with respect to the killing of S. aureus without resistance. To test whether resistant bacteria showed this same effect, Xen36 was subjected to the same conditions as done for Xen29. Interestingly, both Peptoid 1 and Peptoid 1-C13_4mer_ were able to kill Xen36 to background levels of luminescence at concentrations of greater than and equal to 1.5 μM and 6.25 μM within 30 min, respectively, similar to their effect in Xen29 (Fig. 8a-b). Peptoid 1-11_mer_ was far less effective in Xen36 and was unable to fully kill Xen36 to background levels by 30 min (Fig. 8c). Both LL-37 and daptomycin were unable to kill Xen36 at any concentrations under 50 μM, despite incubation for 210 min (Fig. 8d, f). Vancomycin was unable to reach background levels of luminescence at all tested concentrations by 30 min, however, all concentrations were able to get close to background levels of luminescence by 210 min (Fig. 8e). CFU data seen in Fig. 2 confirmed that there was complete killing of S. aureus for the tested peptoids and antibiotics.

## DISCUSSION

The peptoids described in this study show the potential as novel therapeutic agents. This is exemplified by the low concentrations needed for complete killing of both MSSA and MRSA strains, especially by Peptoid 1 but also by Peptoid 1-C13_4mer_. Interestingly, it appears that the TFA salt form of Peptoid 1 was more effective for MRSA 252 but the chloride salt was better for the bioluminescent Xen36 strain. For both Peptoid 1-C13_4mer_ and Peptoid 1-11_mer_, improvements in MICs were seen for the chloride salt. This difference is likely due to the change of charge of the molecule with respect to each salt; however, further testing is needed to confirm this. TFA ions can also be cytotoxic. Calculation of CFU for both methicillin-resistant strains incubated with peptoid confirmed complete killing at concentrations well below their respective IC_50_. This suggests that treatment of infections with these peptoids will be safe enough to prevent harmful side effects.

While killing planktonic bacteria is necessary to clear infections in patients, S. aureus is also known to form biofilms, especially in hospital settings (30). As such, we conducted experiments to determine the ability of these peptoids to prevent the formation of biofilms and to accomplish the detachment of biofilms. Interestingly, these peptoids were the only treatment that could completely prevent formation of or detachment of biofilms. Peptoid 1 once again showed the best prevention and detachment, doing so at concentrations around 1.6 μM for MRSA. It is unknown exactly how these peptoids are able to prevent biofilm formation and detach biofilms, however, it is likely related to their ability to kill planktonic S. aureus and possibly to interaction with biofilm matrix elements.

Since most infections of S. aureus are seen in wounds, we sought out to identify whether topical application of Peptoid 1 and Peptoid 1-C13_4mer_, the most promising candidates, would reduce infection in a murine incision wound model. Even though the peptoids previously have been shown to be active against S. aureus in an *in vivo* murine abscess the peptoids in this case were administered via injection rather than being applied topically to the wound. Topical administration is preferred for wound infections over subcutaneous injections as it is less invasive, and easier for patient self-administration. After 8 days postinfection, there was greater than 99% reduction of bioluminescence for Peptoid 1-treated mice, suggesting clearance in these mice. Control mice, on the other hand, still had significant amounts of bacterial bioluminescence present. There was some reduction compared to initial dosage in the control mice, likely due to the innate immune system. Additionally, control mice did not clear the infection in the allotted time frame, suggesting that the innate immune response alone is not enough to eliminate the bacteria. It should be noted that despite the high concentration of peptoid used in these studies, the mice did not show any signs of visible toxicity. This may be due to the inefficiency of the peptoid to penetrate through the wound based on topical application. This does, however, suggest that peptoids likely will have minimal side effects *in vivo*. This result is consistent with previous studies demonstrating nontoxicity of peptoids *in vivo* (28). Since these data were all collected using bioluminescent imaging (BLI), it allows for fewer mice to be necessary, as it monitors flux in real-time. This is a distinct advantage over traditionally used methods as it does not require sacrifice of mice until the experiment has concluded. The use of BLI revealed inadequacies of current protocols for wound models. Many wound models suggest putting controls and treatment on the same mouse, however, results from our study show migration of infection within a single mouse. This would suggest that the use of one mouse may not necessarily mimic the bio-physiological characteristics of a health and compromised individual simultaneously. In addition to this, lesion size does not necessarily correlate to resolution of infected wounds, as bacteria often present in the deeper layers of tissue and travel to areas away from the initial infection site. Currently, the endpoints of wound healing are mainly measured by the closure of the wound along with absence of the pathogen from the initial wound site. Since bacteria can penetrate deeper into tissues, it is necessary to monitor infection throughout the body to prevent reemergence of infection. Future studies to determine the length of time needed after traditionally used measurements are needed for full clearance.

In this study, we showed the efficacy of antimicrobial peptoids against S. aureus, including resistant strains, both *in vitro* and *in vivo*. We were able to show that these peptoids are fast and efficient killers, doing so in very few minutes. Future studies on improving topical treatments may prove even more valuable in decreasing the amount of peptoid needed as a treatment. Furthermore, the effect of peptoid treatment on immunochemical parameters, including inflammation markers should be assessed, in order to better understand in depth, the effect of peptoids on infection control.

## MATERIALS AND METHODS

### Peptoid synthesis.

Peptoid synthesis was carried out using an ABI 433A (Applied Biosystems, Inc., Foster City, CA) peptide synthesizer and a Symphony X (Gyros Protein Technologies, Tucson, AZ) peptide synthesizer located at the Molecular Foundry in the Lawrence Berkeley National Laboratory, Berkeley, CA. Peptoids were synthesized on a Rink amide MBHA resin (EMD Biosciences, Gibbstown, NJ). All reagents were purchased from Sigma-Aldrich (St. Louis, MO). Synthesis followed the submonomer protocol from Zuckermann, et al. (31). Peptoids were cleaved from the resin by treating with trifluoroacetic acid (TFA):triisopropylsilane:water (95:2.5:2.5 volume ratio) for 10 min. A C18 column in a reversed-phase high performance liquid chromatography (HPLC) system (Waters Corporation, Milford, MA) was used for purification with a linear acetonitrile and water gradient with a compound purity greater than 95% as measured by analytical reverse-phased HPLC. Confirmation of the peptoid synthesis was determined using electrospray ionization mass spectrometry.

### *In vitro* antimicrobial activity against bioluminescent S. aureus.

Bioluminescent Xen 29 and Xen 36 methicillin-susceptible S. aureus (MSSA) strains (obtained from Xenogen Corp. now part of PerkinElmer, Waltham, MA) were grown at 37°C in Luria-Bertani (LB) broth with shaking overnight with 200 μM kanamycin. 2:1 serially diluted concentrations of 50 μL peptoids (200 μM – 1.6 μM) were added to a 96-well plate (black plate with clear bottom). Then, 1 × 10^6^ CFU in 50 μL of the overnight culture was added into the 96-well plate to create a 100 μL 1:1 peptoid: bacterial solution.

The kinetics of the cell killing was monitored by bioluminescent imaging using an IVIS200 (PerkinElmer) and data were analyzed using the Living Image software (PerkinElmer). The luminescence signal was measured every 5 min for 80 min. The MIC was determined after 24 h. All experiments were performed in triplicate and data were analyzed in the GraphPad Prism software (La Jolla, CA).

### Antimicrobial activity of compounds against MRSA.

The methicillin-resistant S. aureus (MRSA) strain used was MRSA 252, a hospital-acquired strain isolated in the United Kingdom. MRSA252 was grown at 37°C in Luria-Bertani broth (LB, BD Bioscience, San Jose, CA). The MIC of the antimicrobials was determined from a range of 0–100 μM by a serial dilution in polypropylene 96-well microtiter plates (BD Bioscience) in accordance with CLSI M7-A6 protocols. To 50 μL of antimicrobial solution (prepared using 2:1 serial dilutions), 50 μL of bacterial solution (1 × 10^6^ CFU/mL) prepared in LB was added and incubated at 37°C for 16 h. MIC was defined as the concentration at which no visible growth was seen when incubated at 37°C for 16 h (h). Bacterial count (CFU/mL) of each strain was determined by OD600. Samples were also plated in LB broth and incubated at 37°C for 24 h in order to determine whether there was complete killing.

### *In vitro* biofilm formation assay.

In a 125 mL Erlenmeyer flask, 400 μL of Staph/MRSA inoculum (1 × 10^6^ CFU/mL), prepared in LB and allowed to grow at 37°C, was added to 40 μL of antimicrobial solution (prepared by 2:1 serial dilutions in TSB). After 24 h, cells were stained by addition of 10 μL (μL) of a 0.5% (wt/vol) crystal violet (CV) solution. Following an incubation of 20 min (min) at room temperature, the supernatant (150 μL) was removed, and wells were washed three times with 200 μL double distilled water, followed by three times blotting on the paper towel, to remove planktonic, unattached, and weakly attached cells. 200 μL of 95% (vol/vol) ethanol was added to dissolve the CV stain. The percentage of biofilm formation was determined by measuring the UV absorbance of CV stain at λ = 600 nm using a microtiter plate.

### *In vitro* biofilm detachment assay.

The detachment assay was performed by pregrowing Staph/MRSA biofilms in LB without the presence of an antibiotic in 96-well plates at 37°C for 24 h. Planktonic, unattached, and weakly attached cells were removed by washing the biofilms three times with LB. To the test wells, 50 μL of antimicrobial solution (prepared by 2:1 serial dilutions in LB) and 50 μL LB were added. After 24 h at 37°C, biofilm reduction was determined by following the CV staining process and measuring the UV absorbance of CV stain at λ = 600 nm using a plate reader.

### Cytotoxicity of peptoids using MTS assay.

Peptoid cytotoxicity has been extensively studied by Huang and Czyzeweski (26, 28) and their protocol was followed. J774A.1 macrophages and 3T3 mouse fibroblast cells were cultured and then exposed to peptoid for 4 h in an (3-(4,5-dimethylthiazol-2-yl)-5-(3-carboxymethoxyphenyl)-2-(4-sulfophenyl)-2H-tetrazolium) assay (MTS) to determine cytotoxicity. Briefly, the cells were cultured in Dulbecco’s Modified Eagle’s Media and then added to a 2:1 serially diluted peptoid solution plate in Hanks’ balanced salt solution. Then 20 mL of the CellTiter 96 Aqueous Non-Radioactive cell proliferation assay (Promega) reagent which contains a tetrazolium compound, [3-(4,5-dimethylthiazol-2-yl)-5-(3-carboxymethoxyphenyl)-2-(4-sulfophenyl)-2H-tetrazolium, inner salt; MTS(a)], was added to each well and cells were further incubated for 2 h to metabolize. After which, the absorbance was measured at 490 nm using a plate reader. Experiments were performed in triplicate.

### Murine incision wound model.

Female CD1 mice were obtained from Jackson Laboratory (Bar Harbor, ME) and, on day 0, anesthetized using isoflurane, hair on the dorsal side was shaved, and the skin was sterilized with an alcohol swab. A 1 cm long incision was made with scissors on the preshaved dorsal surface of the mice. A subcutaneous pocket was then produced using the end of the scissors and 1 × 10^6^ CFU of the bioluminescent S. aureus bacteria in 20 μL was injected into the pocket with a pipette. The wound was then sealed with Vetbond veterinary adhesive. The infection was always subcutaneous, and we were careful not to cause any bleeding. Bacterial growth over time was tracked and quantitated by BLI, using Living Image Software (Perkin Elmer) according to the instructions of the manufacturer. In order to test the antibacterial activity of the peptoids, after the establishment of the infection at day 1, wounds on half of the mice (*n* = 4) were treated topically with 60 μL of peptoid in water or PBS, while wounds on the remaining mice were treated with 60 μL of the control solution (water or PBS). After treatments, the mice continued to remain anesthetized for up to half hour until the treatment solution dried. From days 1 to 7, the mice were imaged daily for growth of infection before the treatment was applied. Mice were randomly selected and divided into groups based on treatment. Many treatments were tested, including water (control), PBS (control), Peptoid 1 in PBS or water (with concentrations varying from 100–800 μM), and Peptoid 1-C13_4mer_ in PBS or water (with concentrations varying from 100–800 μM). The kinetics of the cell killing was monitored by BLI performed using an IVIS 200 (PerkinElmer). The mice were anesthetized by isoflurane and bioluminescence was recorded for 60 s (s) at medium binning, and analyzed using the Living Image Software, with equal region of interest measurements. To determine the pervasiveness of the infection in untreated wounds, after imaging wounds from the control mice treated with PBS, animals were sacrificed and a tissue section that included the wound area was excised. The section was placed in the IVIS and bioluminescence was measured, after which the section was flipped upside down in order to image the luminescence from the underside of the wound. In this manner, it could be determined if the bacteria were residing on the surface or beneath the surface of the wound. This study was carried out in accordance with the recommendations in the Guide for the Care and Use for Laboratory Animals of the National Institutes of Health and used a protocol approved by Institutional Animal Care and Use Committee.

### Statistical analyses and animal numbers.

The minimum numbers of animals that would allow statistically significant differences to be observed were used in all experiments. This estimate was based on power analysis using conditions that will allow sensitivity for 2-fold differences at a *P* value of 0.05 and an estimated standard deviation based on our previous studies with similar experimental strategies and imaging technologies. Unless stated otherwise, comparison of three or more groups were by ANOVA with the Tukey-Kramer *post hoc* pairwise *t* test. The Student's *t* test was used for pairwise comparisons. *P* values of less than 0.05 were considered significant.
