# Supplementary material for: Efficacy of Cathelicidin-Mimetic Antimicrobial Peptoids against Staphylococcus aureus
Source: Microbiol Spectr. 2022 Apr 25;10(3):e00534-22. doi: 10.1128/spectrum.00534-22 (PMC9241801; doi:10.1128/spectrum.00534-22)
Supplement: SUPPLEMENTAL FILE 1 — Supplemental material. Download spectrum.00534-22-s001.pdf, PDF file, 0.5 MB [file spectrum.00534-22-s001.pdf]

Supplementary Material

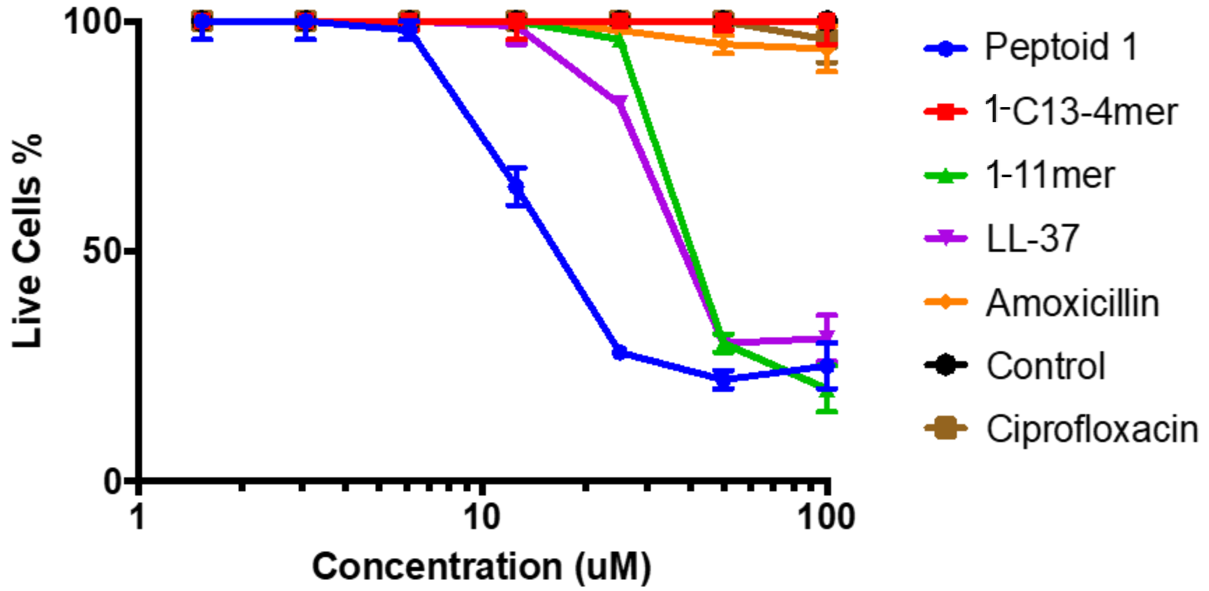

Figure S.1: Cytotoxicity of peptoids against 3T3 cell lines in culture. The median lethal dose (LD_{50}) was determined as the concentration at which 50% of the cells were killed. All data points are represented as means using three replicates. Error bars are represented as ± standard deviation (SD).

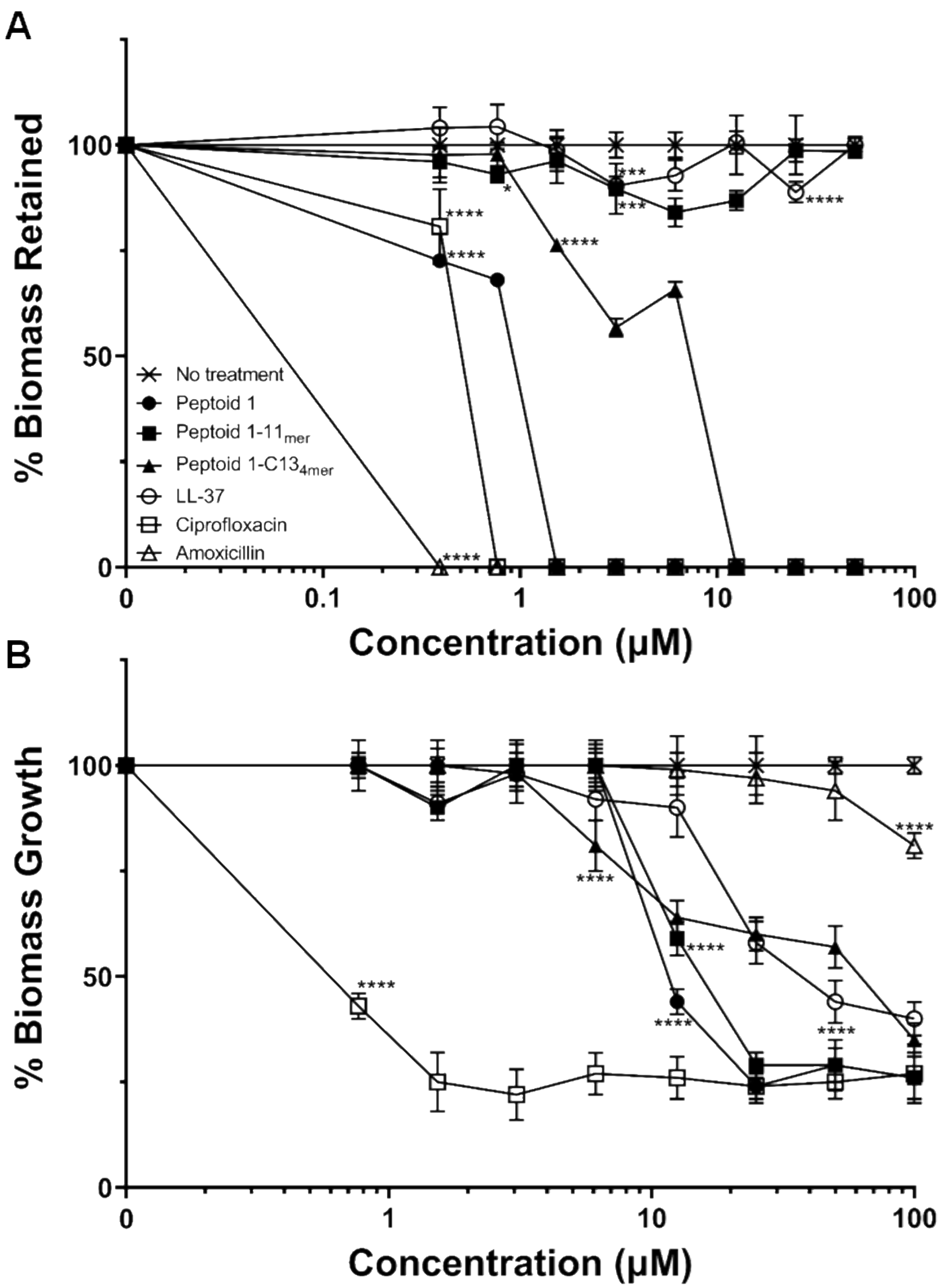

Figure S.2: Measurement of planktonic *Staphylococcus aureus* (**a**) Xen29 and (**b**) Xen36 biofilm formation in LB for 24 hours in the presence of antimicrobial peptoids (AMPs) and conventional antibiotics. Prevention of biofilm formation is plotted as percent biomass analyzed by crystal violet (CV) staining assay. (**a**) Peptoid 1, ciprofloxacin, and amoxicillin were all significant starting at all concentrations tested. Peptoid 1-C13$_{4mer}$ was significant

compared to the no treatment control starting 1.56 µM and up while Peptoid 1-11$_{mer}$ was significant at 0.78 µM and from 3.125 µM to 12.5 µM. LL-37 showed significance from 3.125 µM to 6.25 µM and once again at 25 µM. (**b**) For Xen36, ciprofloxacin showed a significant decrease from the no treatment control for all tested concentrations. Peptoid 1-C13$_{4mer}$ showed significance from 6.25 µM onward, while Peptoid 1 and Peptoid 1-11$_{mer}$ showed significance starting at 12.5 µM. LL-37 showed significance from 25 µM onward, while amoxicillin only showed significance at 100 µM. All data points are represented as means using three replicates. Error bars are represented as ± standard deviation (SD). Statistics were performed using 2-way ANOVA, comparing each antimicrobial to the no treatment control. P values are: < 0.0001 = ****, between 0.0001 and 0.001 = ***, between 0.001 and 0.01 = **, and between 0.01 and 0.05 = *.

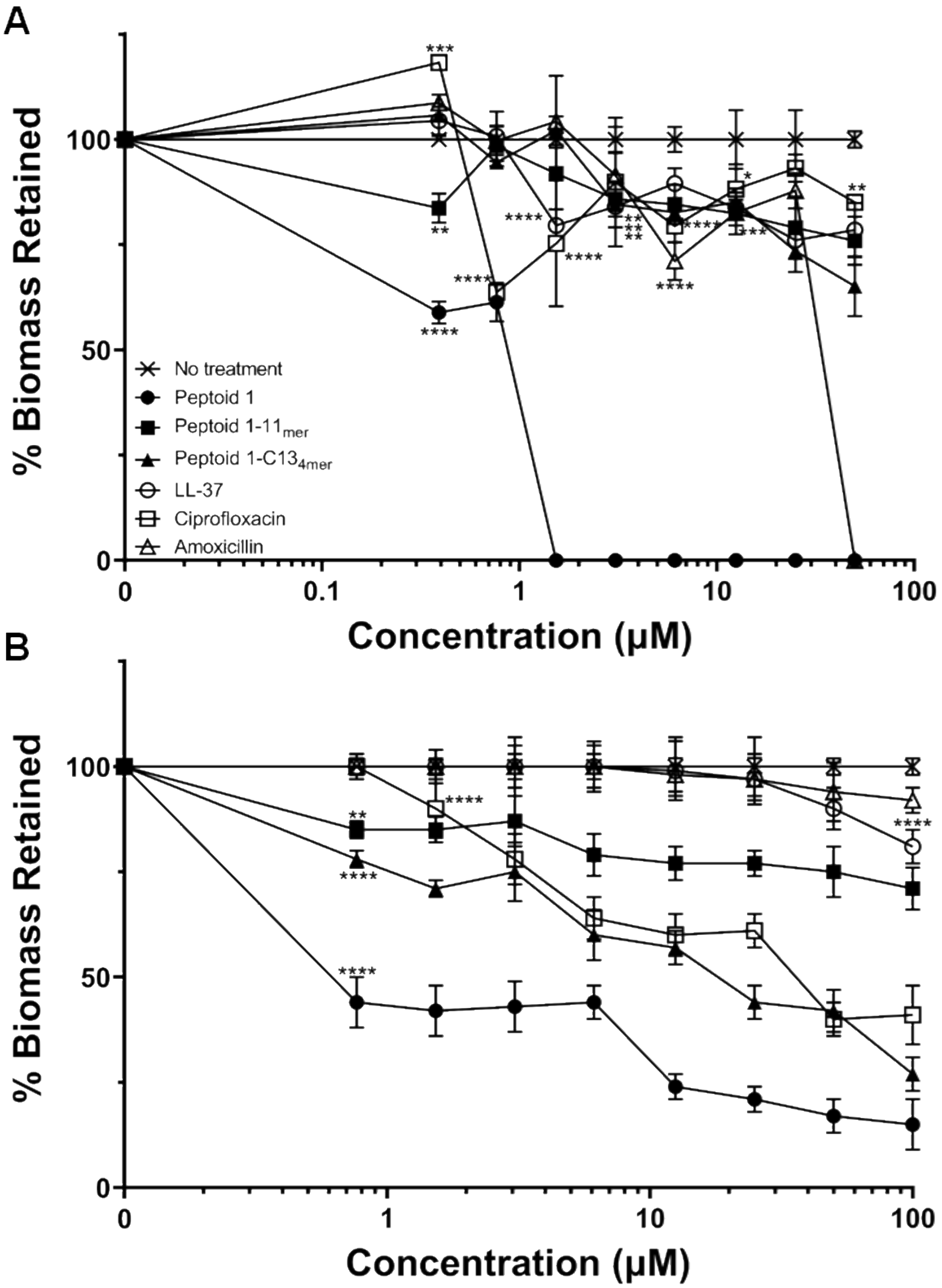

Figure S.3: Measurement of (**a**) Xen29 and (**b**) Xen36 biofilm detachment in LB for 24 hours by antimicrobial peptoids (AMPs) and conventional antibiotics. Detachment is plotted as percent biomass of established biofilms after treatment with antimicrobials. (**a**) Peptoid 1 showed a significant decrease for all tested concentrations compared to the no treatment control. Peptoid 1-11_{mer} showed a significant decrease at 0.39 µM, however,

didn't show significance again until 3.125 µM, where both Peptoid 1-C13$_{4mer}$ and it showed significance onwards. LL-37 showed significance initially from 1.56 µM to 3.125 µM and once again from 12.5 µM onward. Ciprofloxacin showed an initial increase compared to the no treatment control at 0.39 µM, however, decreased at all other concentrations except for 3.125 µM and 25 µM, while amoxicillin showed significance from 6.125 µM onward. (**b**) For Xen 36, Peptoid 1, Peptoid 1-C13$_{4mer}$, and Peptoid 1-11$_{mer}$ all showed a significant decrease when compared to the no treatment control at all tested concentrations. Ciprofloxacin showed a significant decrease in biomass retained at concentrations above 1.56 µM. Amoxicillin showed no significant decrease at all tested concentrations, while LL-37 only showed significance at 100 µM. All data points are represented as means using three replicates. Error bars are represented as ± standard deviation (SD). Statistics were performed using 2-way ANOVA, comparing each antimicrobial to the no treatment control. P values are: < 0.0001 = ****, between 0.0001 and 0.001 = ***, between 0.001 and 0.01 = **, and between 0.01 and 0.05 = *.

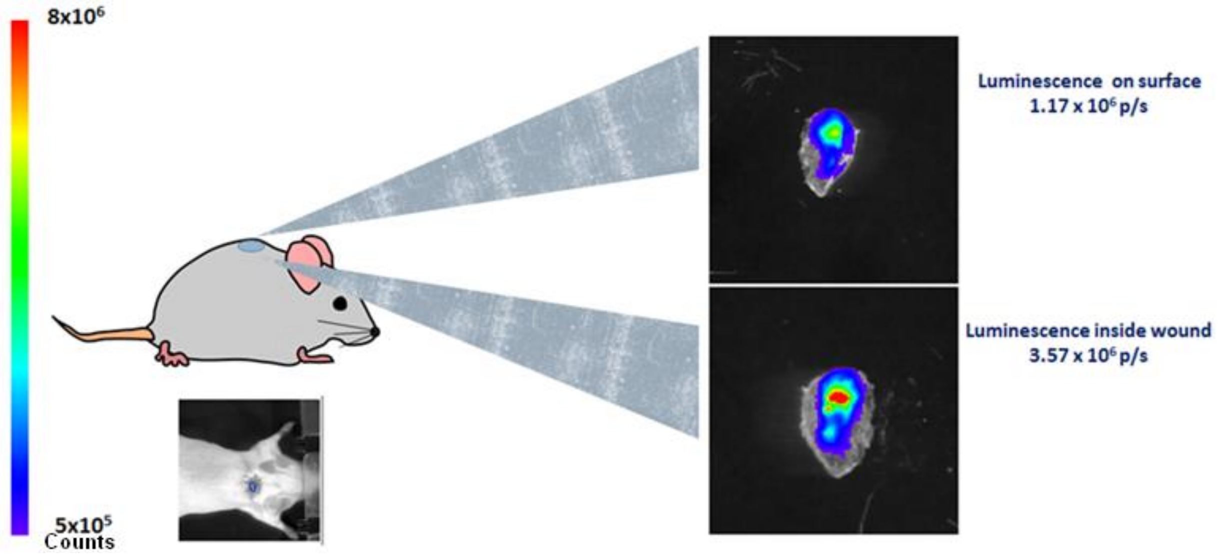

Figure S.4: Bioluminescence of excised wound. Wounds were excised out, imaged from control animals in **Figure 6**, and imaged from both top and bottom. (**a**) Top (dorsal) side image with total bioluminescence of 1.17 x 10^6 photons per second (p/s). (**b**) Bottom (ventral) side image with total bioluminescence of 3.57 x 10^6 p/s.

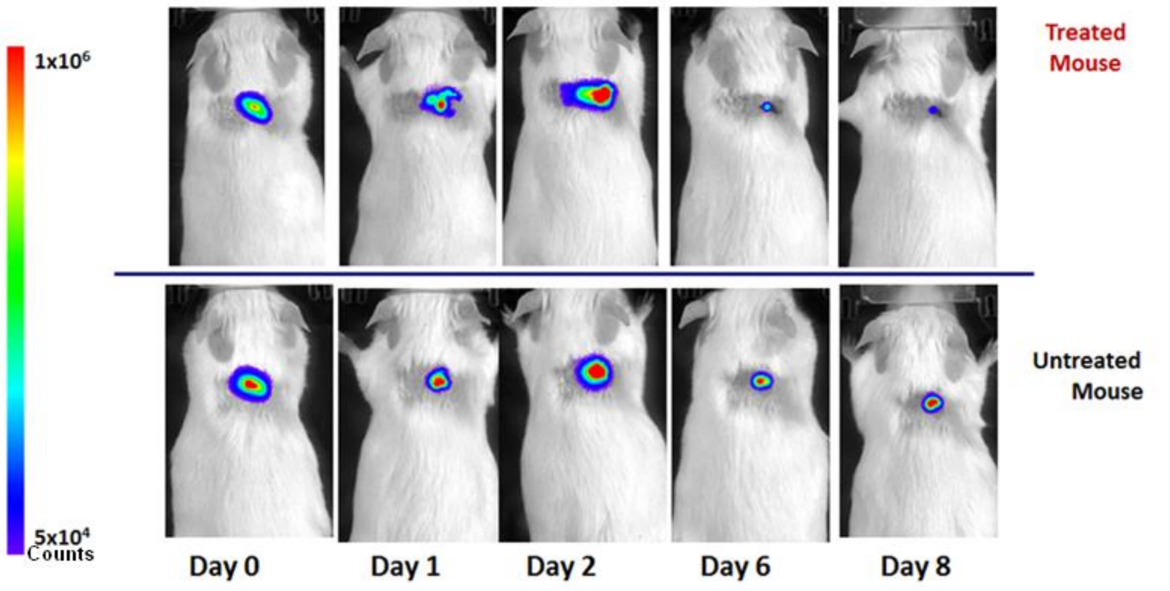

Figure S.5: *In vivo* bioluminescence imaging. Bioluminescent *Staphylococcus aureus*, Xen36, was injected into the incision wounds. Wounds on mice treated with 800 µM Peptoid 1 in water, showed negligible bioluminescence for eight days (mice above line). Control mice treated with water (below line) still showed bioluminescence by Day eight.

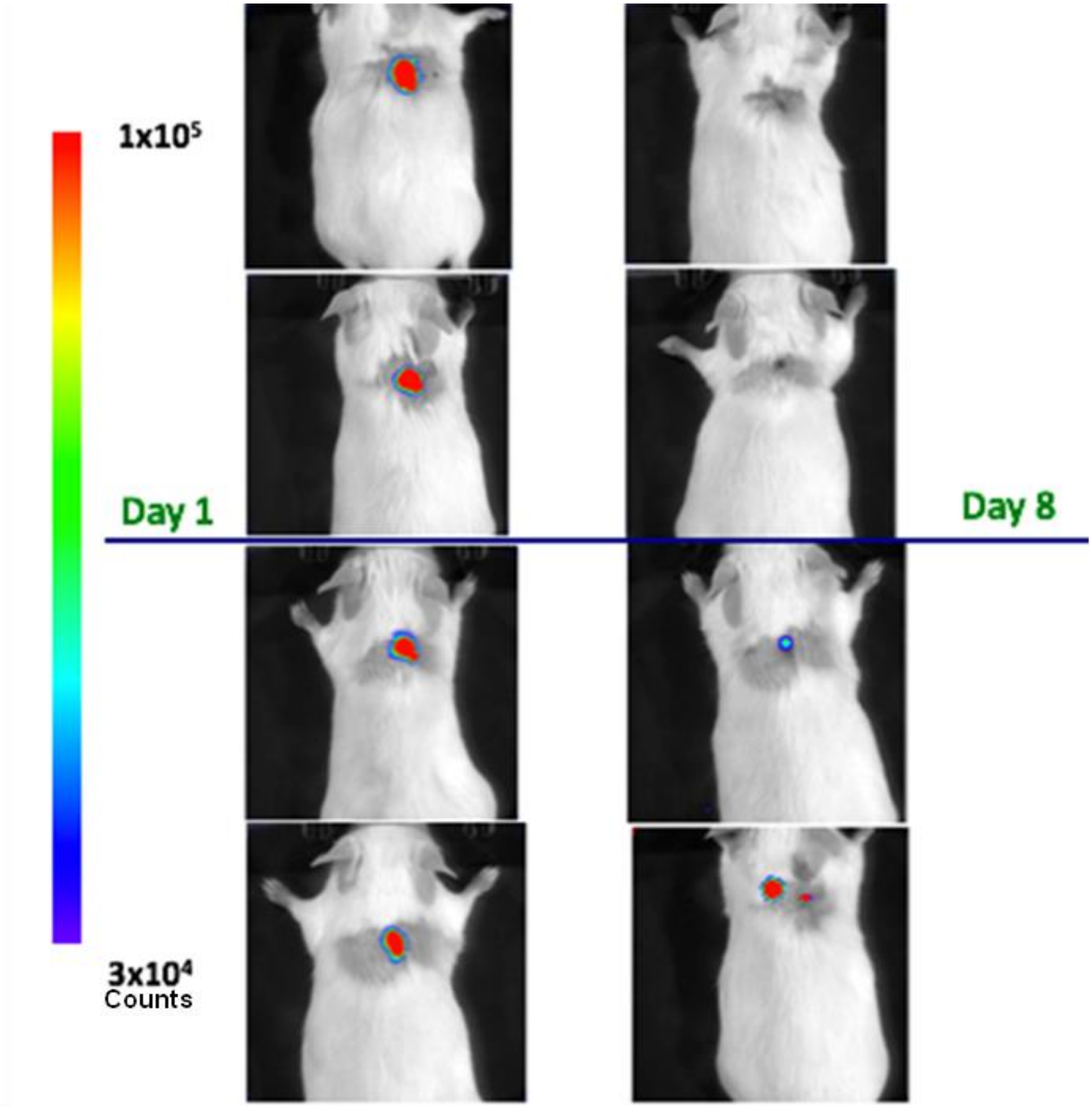

Figure S.6: *In vivo* bioluminescence imaging. Bioluminescent *Staphylococcus aureus*, Xen36, was injected into the incision wounds. Wounds on mice treated with 800 µM Peptoid 1-C13$_{4mer}$ in water (**Above Line**), showed negligible bioluminescence for eight days. Control mice (**Below Line**) treated with water buffer showed bioluminescence at eight days.

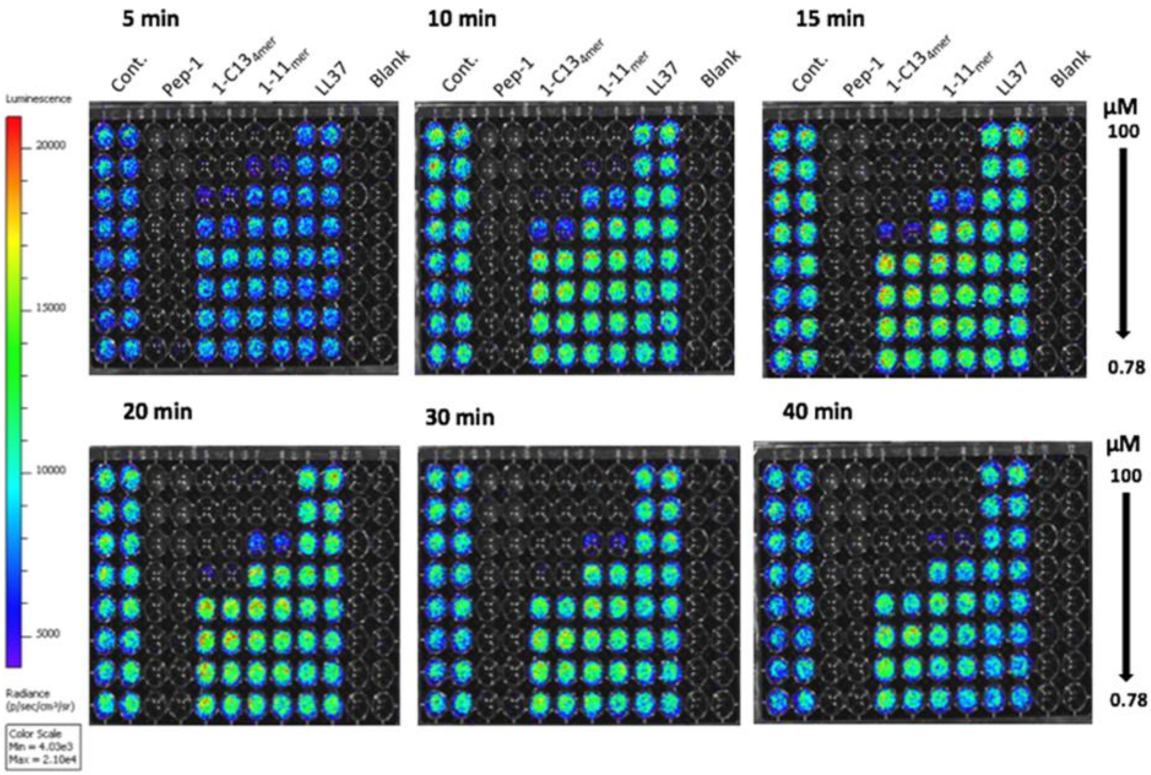

Figure S.7: An example bioluminescent assay after inoculation with Xen29 and incubation for 5-40 min at 35 ^{o}C. Growth in column one (Just Cells) shows normal growth of the bacterial inoculum. Column eleven and twelve (Media) show no growth as it does not contain bacteria and demonstrates that the media was properly sterilized and handled aseptically. Wells in columns three and four show the inhibitory activity of Peptoid 1, Peptoid 1-C13$_{4mer}$ in columns five and six, and so on.
